# The Action of Glycerine in Nephrolithiasis

**Published:** 1893-09

**Authors:** 


The Action of Glycerine in Nephrolithiasis.—Besides piperazine, which is the best known solvent of concretions of uric acid and its salts, glycerine has attracted attention in recent literature as a remedy in nephrolithiasis. Upon the administration of fifty to one hundred cubic centimeters of glycerine, concrements to the size of a bean have been observed to pass away with the urine in patients suffering from nephrolithiasis fourteen to fifteen hours after taking the drug, the urine also containing a remarkable amount of mucus. Two or three hours after the drug has been taken, pains occur with great regularity in the region of the suspected kidney. In order to explain this action of glycerine, A. Hermann has made experiments, which have been published in the *Prag. Med. Wochen.*, from which the following may be deducted :

The largest part of the glycerine, taken internally, is secreted unchanged within the next twenty hours with the urine, and the latter is neither quantitatively nor qualitatively changed, excepting that it becomes slippery. The solving power of glycerine for concrements is extremely small, even at the boiling point. When introduced into the ureter of rabbits by abdominal section, no contraction of the involuntary muscular fibers of the urinary passages takes place. When administered to excess, per os, similar symptoms occur as are observed when a saturated solution of sodium chloride is injected into the veins. The action of the drug can, therefore, only be a mechanical one. Glycerine, after entering the blood, withdraws a large amount of water from the tissues, which passes through the kidneys, the mucus in the uriniferous tubules shrinks in consequence of the withdrawal of water by the glycerine, and is thereby loosened and with the concrements washed away by the slippery urine.—*Medical Review.*

